# Measuring IgA Anti-β2-Glycoprotein I and IgG/IgA Anti-Domain I Antibodies Adds Value to Current Serological Assays for the Antiphospholipid Syndrome

**DOI:** 10.1371/journal.pone.0156407

**Published:** 2016-06-02

**Authors:** Charis Pericleous, Isabel Ferreira, Orietta Borghi, Francesca Pregnolato, Thomas McDonnell, Acely Garza-Garcia, Paul Driscoll, Silvia Pierangeli, David Isenberg, Yiannis Ioannou, Ian Giles, Pier Luigi Meroni, Anisur Rahman

**Affiliations:** 1 Centre for Rheumatology Research, Division of Medicine, University College London, London, United Kingdom; 2 Laboratory of Immuno-rheumatology, IRCCS Istituto Auxologico Italiano, Milan, Italy; 3 Structural Biology, MRC National Institute for Medical Research, London, United Kingdom; 4 Division of Rheumatology, Department of Internal Medicine, University of Texas Medical Branch, Galveston, Texas, United States of America; 5 Arthritis Research UK Centre for Adolescent Rheumatology, UCL Hospital and Great Ormond Street Hospital, London, United Kingdom; Nippon Medical School Graduate School of Medicine, JAPAN

## Abstract

**Introduction:**

Currently available clinical assays to detect antiphospholipid antibodies (aPL) test for IgG and IgM antibodies to cardiolipin (aCL) and β_2_-glycoprotein I (aβ_2_GPI). It has been suggested that testing for IgA aPL and for antibodies to Domain I (DI), which carries the key antigenic epitopes of β_2_GPI, could add value to these current tests. We performed an observational, multicenter cohort study to evaluate the utility of IgG, IgM and IgA assays to each of CL, β_2_GPI and DI in APS.

**Methods:**

Serum from 230 patients with APS (n = 111), SLE but not APS (n = 119), and 200 healthy controls were tested for IgG, IgM and IgA aCL, aβ_2_GPI and aDI activity. Patients with APS were further classified into thrombotic or obstetric APS. Logistic regression and receiver operator characteristic analyses were employed to compare results from the nine different assays.

**Results:**

All assays displayed good specificity for APS; IgG aCL and IgG aβ_2_GPI assays however, had the highest sensitivity. Testing positive for IgA aβ_2_GPI resulted in a higher hazard ratio for APS compared to IgM aβ_2_GPI. Positive IgG, IgM or IgA aDI were all associated with APS, and in subjects positive for aCL and/or aβ_2_GPI, the presence of aDI raised the hazard ratio for APS by 3–5 fold. IgG aCL, aβ_2_GPI, aDI and IgA aDI were associated with thrombotic but not obstetric complications in patients with APS.

**Conclusion:**

Measuring IgG aDI and IgA aβ_2_GPI and aDI may be useful in the management of patients with APS, particularly thrombotic APS.

## Introduction

In clinical practice three tests are used to detect antiphospholipid antibodies (aPL), the serological hallmark of antiphospholipid syndrome (APS), a condition characterised particularly by vascular thrombosis (VT) and pregnancy morbidity (PM) [1]. Two of these tests are enzyme-linked immunosorbent assays (ELISAs) that measure anti-cardiolipin (CL, aCL) and anti-β_2_-glycoprotein I (aβ_2_GPI) aPL; the third is a functional clotting assay for lupus anticoagulant (LA). The ELISAs measure IgG and IgM aPL, while the LA test does not discriminate between antibody isotypes [1]. New, additional laboratory tests for APS may have benefits of easier standardisation and better prognostic value in asymptomatic aPL carriers, or for determining risk of recurrence of VT and/or PM in patients already diagnosed with APS. Proposed new tests include assays that measure IgA aPL and autoantibodies against domain I of β_2_GPI (DI) [2, 3].

In comparison to IgG and IgM aPL, IgA aPL have been less-studied and are not included in standard serological tests for APS. Both IgA aCL and IgA aβ_2_GPI have yet to be proven specific for APS, as they are also reported to be elevated in patients with systemic lupus erythematosus (SLE) (with or without APS). However, isolated positivity for IgA aβ_2_GPI (in patients negative for IgG/IgM aCL/aβ_2_GPI and LA) is associated with both VT and PM [4] and IgA aβ_2_GPI have been shown to be prothrombotic *in vivo* [5].

The antibodies for which there is clearest evidence of a causal link to development of both thrombotic and obstetric complications in APS are IgG antibodies that can be detected either by binding to CL in the presence of β_2_GPI (IgG aCL) or by binding to β_2_GPI itself (IgG aβ_2_GPI) [6–9]. β_2_GPI, a 50kDa plasma glycoprotein of five domains (DI-DV), circulates primarily in a biochemically reduced state [10] in which DI interacts with DV to form a closed circular β_2_GPI structure. Upon binding to anionic phospholipids on cell membranes via DV, β_2_GPI changes conformation to an open fishhook structure, exposing DI [11, 12]. Antibodies directed against all individual domains of β_2_GPI have been reported, of which IgG anti-DI antibodies (aDI) are most closely linked to the presence of APS [13–15]. IgG aDI titres are elevated in patients with APS compared to disease and healthy controls [16–22], and both affinity-purified IgG aDI from APS serum [23] and a human monoclonal IgG aPL that binds DI (IS4) [24] are prothrombotic *in vivo* [25, 26]. In the same mouse model, recombinant human DI abrogates aPL-induced thrombosis [27]. In two different *in vivo* models, a human monoclonal IgG aDI increases thrombosis and pregnancy loss [28]. Moreover, mice immunised with human or murine β_2_GPI in the presence of CL vesicles, or with human DI, develop aβ_2_GPI and aDI; whilst immunisation with human DII-V or β_2_GPI alone does not induce production of these antibodies [29]. In light of these studies, there is increasing interest in validating assays to measure IgG aDI reliably and to assess their importance in APS.

The significance, if any, of IgM aPL against any of the five β_2_GPI domains is unclear—de Laat and colleagues reported that IgM aDI were no better associated with VT than IgM aβ_2_GPI [19]. For IgA aPL, two studies detected IgA aPL against DIV-V in over 50% of patients with IgA aβ_2_GPI [5, 30, 31], while the importance of IgA aDI to the pathogenesis of APS is unknown. One study used β_2_GPI domain-deleted mutants to inhibit IgA aPL from binding to β_2_GPI—only mutants containing DIV-V had inhibitory ability, while deletion of DI did not affect binding [32].

Given that both IgA aPL and IgG aDI are considered attractive candidates for new diagnostic tests in APS we designed this study to assess and compare the strength of association of circulating ‘classical’ IgG and IgM aCL and aβ_2_GPI, IgA aCL and aβ_2_GPI, and IgG, IgM and IgA aDI with APS and APS-related clinical manifestations.

## Patients and Methods

### Patients and controls

Sera from (n = 111) patients with APS—as defined by revised Sapporo criteria [1], (n = 119) patients with systemic lupus erythematosus (SLE) with or without aPL (fulfilling ACR SLE criteria [33, 34]) in the absence of APS—were collected by informed consent from institutions in UK, Italy and USA involved in this study. Ethical approval for this study was granted by the London-Hampstead Research Ethics Committee (reference 12/LO/0373). Sera from (n = 200) healthy controls (HC) were obtained as part of the Health Survey for England (HSE) 2006 [35] and provided to us by the Health and Social Care Information Centre together with anonymised data on age, gender, ethnicity and confirmation that they had no long-term illness or history of cardiovascular disease. Demographics for APS, SLE and HC subjects are listed in Table 1.

**Table 1:** Demographic and clinical profile of subjects.

	APS	SLE	HC
**No. of subjects (F:M)**	111 (93:18)	119 (107:12)	200 (106:94)
**Mean age (SD)**	44.0 (10.9)	37.9 (13.0)*	44.1 (13.0)
**Ethnicity**^†^	3A; 4A/C; 101C; 2H; 1M	25A; 23A/C; 68C; 3M	23A; 13A/C; 164C
***Clinical manifestations*:**			
**Thrombosis**^‡^	70	-	-
**Arterial**^§^	46	-	-
**Venous**^§^	34	-	-
**Pregnancy morbidity** ^**‡**^	61	-	-
**≤10 weeks’ gestation∞**	27	-	-
**>10 weeks’ gestation∞**	50	-	-
**SLE-associated APS**	26	-	-
**Catastrophic APS**	3	-	-
**Lupus anticoagulant**	87	28	-
***Treatments*:**			
**Oral anticoagulants**	55	1	-
**Heparin**	17	-	-
**Antiplatelet agents**	52	3	-
**DMARDs**	34	109	-
**Oral steroids ≤5mg/day**	15	40	-
**Oral steroids >5mg/day**	1	39	-
**Rituximab**	3	25	-

* SLE group was significantly younger compared to both APS and HC (p<0.05).

^†^ A, Asian; A/C, African-Caribbean; C, Caucasian; H, Hispanic; M, Mixed.

^‡^ 20 patients have a history of both thrombosis and pregnancy morbidity.

^§^ 10 patients have a history of both arterial and venous thrombotic events.

^∞^16 patients have a history of both early and late stage pregnancy morbidity.

The clinical history of patients with APS (n = 111), summarised in Table 1, was recorded in accordance with APS classification criteria [1]. The majority (n = 70, 63%) had a history of VT. Of 93 women with APS, 61 had a history of PM and 20 of those had also suffered at least one thrombotic episode. Three patients had catastrophic APS (two female, one male). LA was measured at each patient’s home institution clinical laboratory. Treatments at the time of sampling were recorded for patients with APS and SLE (Table 1).

### Direct binding assays to detect aPL

For all assays, half of a 96-well plate was coated with antigen while the other half was treated with buffer alone. Net OD was obtained by subtracting the OD of the non-coated half from the OD of the antigen-coated half. The following anti-human horseradish peroxidase conjugates were used: IgG—A6029, Sigma UK; IgM—A6907, Sigma UK; IgA—ab97215, Abcam UK.

Sera were tested in duplicate at 1:50 dilution in the first instance; sera with activity above the highest calibrator were further diluted to determine exact activity. In all assays, activity of the highest calibrator corresponded to a net OD of 1.2–1.5. Inter- and intra-plate variations were <10% for all nine assays.

### Detecting IgG, IgM and IgA aCL

We measured aCL as per consensus criteria protocols [36] and as previously described [37] using commercially sourced calibrators (Louisville APL Diagnostics, TX, USA). Activity was defined as IgG/IgM/IgA phospholipid units (GPLU/MPLU/APLU respectively). Serum activity was calculated as per manufacturer’s instructions. The calibrators’ activity ranges were 16-96GPLU; 16-96MPLU; and 2.7-120APLU.

### Detecting IgG, IgM and IgA aβ_2_GPI and aDI

We measured aβ_2_GPI activity as previously described [37]. aDI were measured in the same manner; instead of human β_2_GPI, plates were coated with human recombinant DI, expressed in-house in bacteria and refolded to adopt its physiological conformation [24, 38].

In-house calibrators were used for aβ_2_GPI and aDI assays. For IgG assays, affinity purified IgG aDI isolated from the serum of a patient with APS was used. For IgM and IgA assays, serum from a different patient with high IgM and IgA aβ_2_GPI & aDI activity was used. All calibrators were serially diluted to obtain a standard curve, and arbitrary activity units were assigned to each point. aβ_2_GPI and aDI activity were defined as IgG/IgM/IgA β_2_GPI units (GBU/MBU/ABU respectively) and DI units (GDIU/MDIU/ADIU respectively), and calculated as per aCL assays. For aβ_2_GPI assays, calibrators’ activity ranges were 3-100GBU; 13-100MBU; and 7-100ABU. For aDI, the ranges were 3-100GDIU; 9-100MDIU; and 2-100ADIU.

### Statistical analysis

Logistic regression analysis was employed to determine possible associations between aPL titres and APS (within the entire cohort, n = 430). As we did not test for LA ourselves, we only had robust LA data for APS but not SLE or HC subjects, and thus were unable to determine the strength of association between LA and APS in our cohort.

We additionally determined possible associations between aPL titres and: ‘primary’ versus ‘secondary’ APS; thrombotic versus obstetric APS; LA positivity (within the APS cohort, n = 111, excluding male patients where necessary). *P* values determined significant positive or negative associations. Hazard ratios (HR), or odds ratios, and 95% confidence intervals (95%CI) are reported. A significant association is determined when the 95%CI range excludes 1.0, where values >1.0 denote a positive association.

Receiver operating characteristic (ROC) analysis, performed to assess the discriminatory ability of each aPL test for APS, generated values for: accuracy (area under the curve, where a value of 1 represents a perfect test without false negatives or false positives); specificity (where 100% suggests no false positives); sensitivity (where 100% suggests no false negatives), and positive likelihood ratios (which reflects the proportion of patients who have APS and test positive to the proportion of patients who do not have APS but also test positive).

We performed logistic regression and ROC analyses using Stata10. Correlation tests (to compare different aPL titres of the same isotype), one-way ANOVA and Fisher’s exact tests (to compare age and gender in APS, SLE and HC) were performed in GraphPad Prism 5.

## Results

### IgG aPL are present in a higher proportion of patients with APS than IgM or IgA antibodies and only IgG antibodies are associated with LA positivity

**Fig 1:**
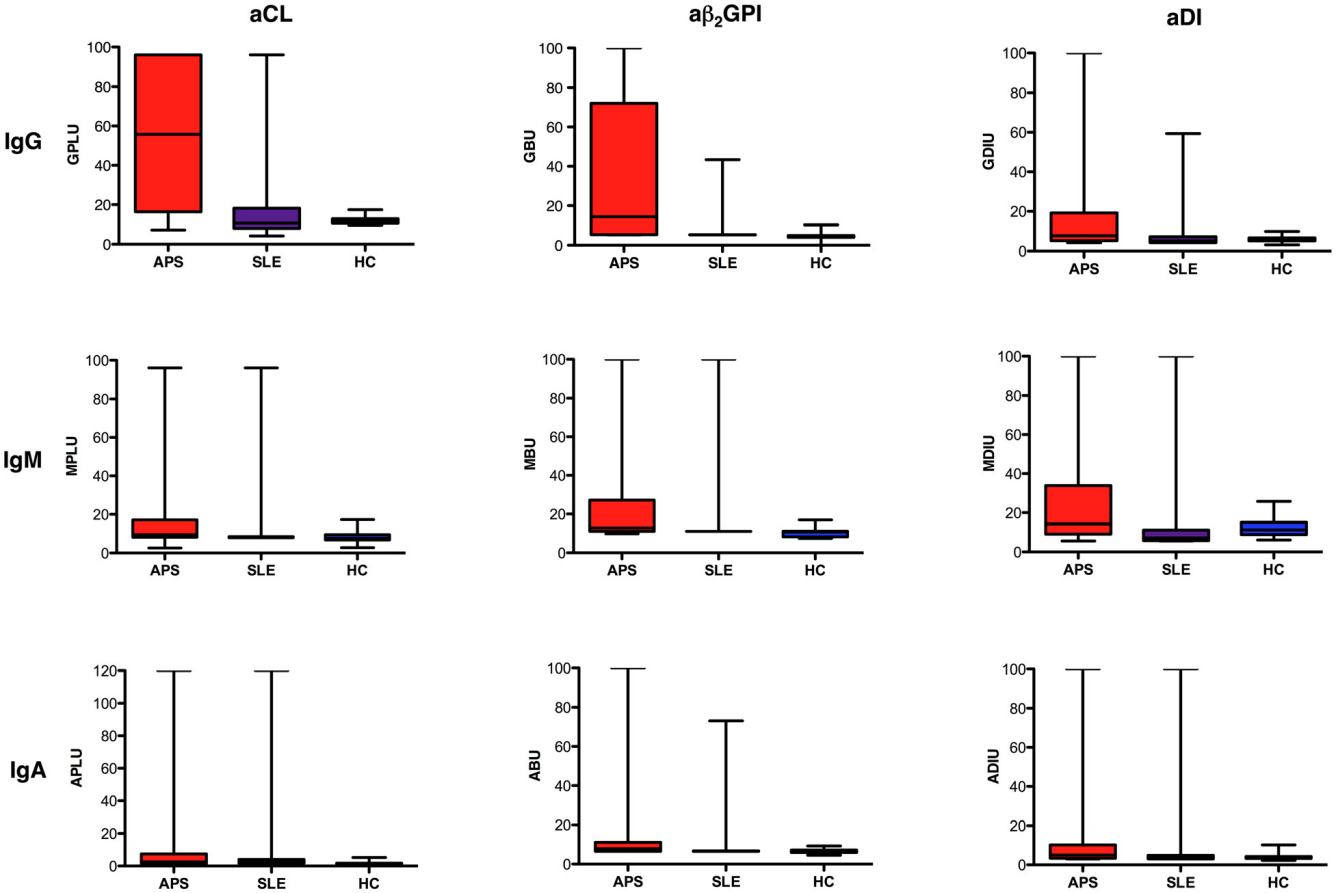
aPL titres in APS, SLE and healthy control (HC) subjects. Sera collected from a total of 111 patients with APS, 119 with SLE (but not APS) and 200 healthy controls were tested in nine aPL assays. Box and whisker plots running from top to bottom depict IgG, IgM and IgA titres of (running from left to right) aCL, aβ_2_GPI and aDI for each subject studied. The black line across data sets denotes mean activity (mean values are listed in Table 1). Abbreviations: GPLU, MPLU, APLU: IgG/IgM/IgA phospholipid units respectively; GBU, MBU, ABU: IgG/IgM/IgA β_2_GPI units respectively; GDIU, MDIU, ADIU: IgG/IgM/IgA DI units respectively.

aPL positivity was defined as titres >99^th^ percentile of the mean activity of our HC cohort. Cut-offs for positivity were determined to be: 17GPLU; 8GBU; 10GDIU for IgG aCL, aβ_2_GPI and aDI respectively, 17MPLU; 16MBU; 21MDIU for IgM aCL, aβ_2_GPI and aDI respectively, and 4APLU; 9ABU; 8ADIU for IgA aCL, aβ_2_GPI and aDI respectively. Mean aPL activity for APS, SLE and HC, and the percentage of subjects from each of these groups that tested positive in each assay, are listed in Table 2. Results from individual subjects for all nine assays are graphically shown in Fig 1.

**Table 2:** aPL activity and percentage of positivity in APS, SLE and HC groups.

	aCL		aβ_2_GPI		aDI	
	Mean titre (SD)	% positive	Mean titre (SD)	% positive	Mean titre (SD)	% positive
***IgG aPL*:**						
**APS (n = 111)**	55.8 (36.5)	74	35.4 (36.6)	65	22.8 (30.9)	41
**SLE (n = 119)**	17.2 (17.6)	30	6.2 (4.1)	8	6.9 (5.9)	11
**HC (n = 200)**	11.9 (1.7)	1	4.6 (1.1)	4	6.0 (1.4)	1
***IgM aPL*:**						
**APS (n = 111)**	18.9 (21.8)	27	28.3 (29.5)	33	25.5 (25.2)	35
**SLE (n = 119)**	11.6 (13.4)	7	14.9 (16.6)	6	11.0 (11.7)	8
**HC (n = 200)**	7.9 (2.8)	1	10.3 (1.8)	1	12.4 (4.5)	5
***IgA aPL*:**						
**APS (n = 111)**	17.1 (34.7)	38	16.2 (23.4)	46	12.5 (21.2)	41
**SLE (n = 119)**	5.9 (16.0)	30	7.9 (7.3)	8	6.4 (16.0)	7
**HC (n = 200)**	1.7 (0.7)	3	6.4 (0.9)	1	4.0 (1.3)	2

Mean aPL titres (standard deviation) for all APS, SLE and HC subjects.

The ideal diagnostic test would be one in which a large proportion of APS and, crucially, only a minority of non-APS subjects would test positive. Comparing the three isotypes, our results show that the IgG aPL assays came closest to approaching this ideal. In patients with APS, positivity for IgG aPL was the most frequent (percentage positivity range 41–74%) and of higher mean titres than IgM or IgA aPL. Interestingly, in patients with APS, IgA aPL were more often positive (38–46%) than IgM aPL (27–35%) (Table 2, Fig 1).

IgG aCL were more prevalent in APS compared to aβ_2_GPI and aDI. Importantly however, IgG aCL were also found in 30% of the SLE group (as previously reported [39]), as were IgA aCL. In fact, for all three isotypes, aβ_2_GPI assays showed the best discrimination between APS and non-APS—no more than 8% of SLE or HC tested positive for aβ_2_GPI of either of the three isotypes. Similarly, aDI were detected in very few SLE or HC (Table 2).

Looking at the group of 111 subjects with APS alone, being positive for IgG aCL was associated with increased likelihood of being LA positive (hazard ratio (HR) 1.9, 95%CI 1.1–3.3, p = 0.017) and this was also true for IgG aβ_2_GPI (HR 1.8, 95%CI 1.1–3.1, p = 0.002) and IgG aDI (HR 2.2, 95%CI 1.1–4.5, p = 0.035). Conversely, positivity for IgM or IgA antibodies to any of these three antigens was not associated with LA (data not shown).

There were no significant differences in any of the nine assays tested between patients with APS but no other autoimmune disease (‘primary’ APS) and patients with SLE-associated (‘secondary’) APS. aPL titres were not associated with age or gender (data not shown).

### ROC and logistic regression analysis indicate that IgG and IgA aβ_2_GPI assays are the best discriminators of APS

**Fig 2:**
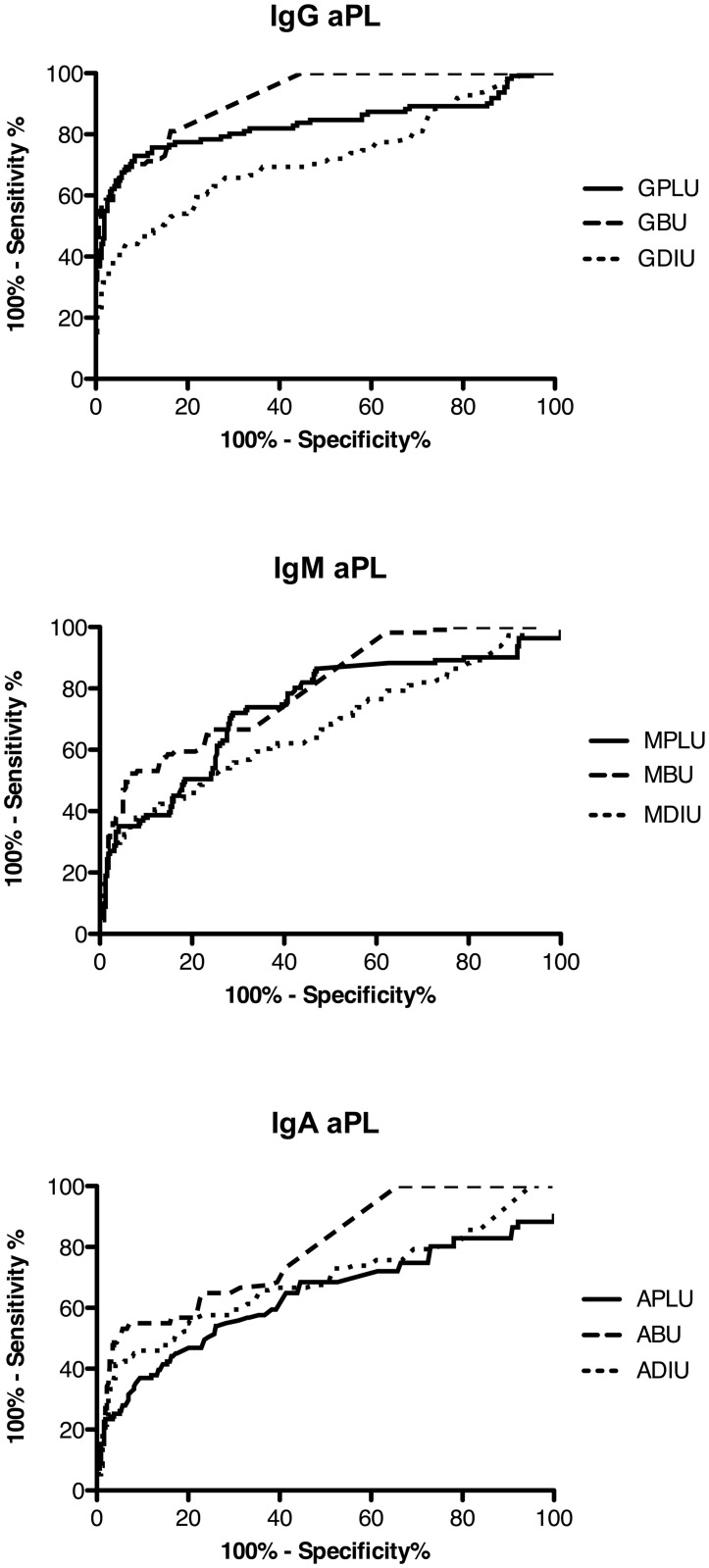
ROC analysis: aβ_2_GPI tests best discriminate for APS. Receiver operating characteristic (ROC) analysis was performed to assess the ability of each of the nine aPL assays to discriminate between APS and non-APS subjects. For all three antibody isotypes, the resulting ROC curves illustrate the superiority of aβ_2_GPI tests compared to aCL and aDI for APS diagnosis (numerical results are listed in Table 3). Abbreviations: GPLU, MPLU, APLU: IgG/IgM/IgA phospholipid units respectively; GBU, MBU, ABU: IgG/IgM/IgA β_2_GPI units respectively; GDIU, MDIU, ADIU: IgG/IgM/IgA DI units respectively.

ROC analysis (Table 3, Fig 2) confirmed that, for all three isotypes, aβ_2_GPI were best associated with APS compared to aCL and aDI, with IgG aβ_2_GPI positivity being the strongest discriminator for APS. For the purposes of this study, we report the specificity and sensitivity of each of the nine aPL for APS at the level of each assay’s calculated cut-off for positivity. As seen in Table 3, all nine assays displayed excellent specificity for APS (~90% or above) but sensitivity was poorer in comparison.

**Table 3:** ROC analysis: discriminatory ability of each aPL test for APS.

	aCL	aβ_2_GPI	aDI
***IgG aPL*:**			
**Area under curve (95% CI)**	0.83 (0.78–0.89)	0.92 (0.89–0.94)	0.72 (0.66–0.78)
**Sensitivity (95% CI)**	72.9 (63.7–80.9)	64.8 (55.2–73.7)	40.5 (31.3–50.3)
**Specificity (95% CI)**	89.7 (85.8–92.8)	95.6 (92.8–97.6)	95.9 (93.1–97.8)
**Likelihood ratio**	7.1	14.8	10.0
***IgM aPL*:**			
**Area under curve (95% CI)**	0.74 (0.68–0.80)	0.80 (0.75–0.85)	0.67 (0.61–0.74)
**Sensitivity (95% CI)**	26.1 (18.3–35.3)	33.3 (24.7–42.9)	33.3 (24.7–42.9)
**Specificity (95% CI)**	97.8 (95.5–99.1)	97.5 (95.1–98.9)	93.7 (90.5–96.1)
**Likelihood ratio**	11.9	13.3	5.3
***IgA aPL*:**			
**Area under curve (95% CI)**	0.64 (0.57–0.71)	0.79 (0.74–0.84)	0.69 (0.62–0.75)
**Sensitivity (95% CI)**	36.9 (28.0–46.6)	40.5 (31.3–50.3)	38.7 (29.6–48.5)
**Specificity (95% CI)**	89.7 (85.8–92.8)	97.2 (94.7–98.7)	95.9 (93.1–97.8)
**Likelihood ratio**	3.6	14.4	9.5

Sensitivity, specificity and likelihood ratios shown are based on the cut off for positivity for each aPL. For all analyses, p≤0.001.

Another way to evaluate each assay’s performance is to compare the likelihood ratios (LR) generated from ROC analysis. We report the positive LR for each assay, which indicates how much the probability of having APS is increased if a subject tests positive. The LR is considered the most clinically relevant for diagnostic tests, and a positive ratio >10 is considered particularly significant [40]. Taking this into account, testing positive for aβ_2_GPI of any of the three isotypes increased the probability of having APS by a factor of >13, followed by IgM aCL (LR 11.9), IgG aDI (LR 10.0) and IgA aDI (LR 9.5) (Table 3).

The results of logistic regression analysis for each assay are shown in Table 4 as the HR that a subject testing positive will have APS compared to a subject testing negative. This analysis was performed in two groups; (a) all subjects (n = 430) and (b) all SLE (n = 145, which included the 119 patients in our SLE/no APS group plus 26 patients from the APS group who also had SLE). The rationale for group (b) is to represent the common clinical scenario of testing patients with SLE to evaluate their risk of developing APS. For both (a) and (b), positivity in each of the nine assays showed a significantly positive HR for APS, with one exception—IgA aCL in the 145 patients with SLE, reflecting the similar prevalence of IgA aCL in both our APS and SLE/no APS patients (38% and 30% respectively, Table 2). Overall, IgG and IgA aβ_2_GPI had the greatest HR for APS (33.4 and 33.9 respectively) (Table 4).

**Table 4:** Regression analysis: hazard ratio for APS.

	aCL	aβ_2_GPI	aDI
***IgG aPL*:**			
**All subjects (n = 430)**	8.6 (5.7–12.9)	33.4 (13.0–86.1)	6.6 (3.8–11.4)
All SLE (n = 145)*	4.2 (2.4–7.5)	9.8 (3.1–31.6)	3.5 (1.8–6.8)
***IgM aPL*:**			
**All subjects (n = 430)**	3.7 (2.4–5.7)	9.2 (4.6–18.4)	3.8 (2.6–5.5)
All SLE (n = 145)*	2.7 (1.3–5.4)	3.2 (1.7–6.2)	2.8 (1.5–4.9)
***IgA aPL*:**			
**All subjects (n = 430)**	2.1 (1.6–2.7)	33.9 (10.5–109.5)	4.5 (2.8–7.1)
All SLE (n = 145)*	1.3 (0.9–1.9)	5.3 (2.1–13.3)	2.2 (1.3–3.7)

Hazard ratio (95% CI) that subjects who test positive in an assay have APS compared to those who test negative in the assay. For all analyses, p≤0.001, with the following exceptions: in ‘All SLE’, IgM aCL p = 0.006; IgA aCL p = 0.15; IgA aDI p = 0.004.

* In the ‘All SLE’ group, 26 of 145 patients had APS.

### IgG, IgM and IgA aDI assays have high specificity but IgG aDI has lower sensitivity than IgG aβ_2_GPI for APS

IgG, IgM and IgA aDI assays showed excellent specificity and similar sensitivity for APS compared to aβ_2_GPI, with the exception of IgG aDI which was less sensitive than both IgG aβ_2_GPI and IgG aCL (Table 3). Positivity for IgG, IgM and IgA aDI was strongly associated with APS to a similar or better level than the corresponding aCL assays, though not as strongly as with aβ_2_GPI (Table 4).

For each immunoglobulin isotype, we noted a strong positive correlation between aDI and aβ_2_GPI titres across the entire cohort of 430 subjects (r = 0.7–0.8 in all cases, p<0.001), however when only APS subjects were considered, the correlation between IgG aDI and aβ_2_GPI dropped to a moderate level (r = 0.6, p<0.001). This discrepancy is because some APS patients had medium-high IgG aβ_2_GPI but low aDI, or vice versa, thus some patients’ reactivity against the whole molecule was different to reactivity against DI.

### Does testing for aDI add value to current diagnostic tests?

The majority of patients testing positive for aDI were also positive for aCL, aβ_2_GPI and LA (Table 5). We therefore next assessed whether the inclusion of a test for aDI positivity would add value in aCL and/or aβ_2_GPI-positive cases. For this purpose, we compared the HR for APS in subjects positive for aCL and/or aβ_2_GPI but not aDI [aCL/aβ_2_GPI+(aDI-)] versus subjects with aDI [aCL/aβ_2_GPI+(aDI+)] (Table 6).

**Table 5:** Triple, double & single positivity per antibody isotype in APS.

	IgG aPL		IgM aPL		IgA aPL	
	All APS (n = 111)*	LA +ve APS (n = 87)^†^	All APS (n = 111)*	LA +ve APS (n = 87)^†^	All APS (n = 111)*	LA +ve APS (n = 87)^†^
**Triple aCL/aβ**_**2**_**GPI/aDI+**	40	36	22	15	27	23
**Double aCL/aβ**_**2**_**GPI+**	26	19	6	4	9	7
**Double aCL/aDI+**	4	2	1	0	1	1
**Double aβ**_**2**_**GPI/aDI+**	1	0	6	6	5	1
**aCL+ only**	12	7	2	2	6	5
**aβ**_**2**_**GPI+ only**	5	2	5	2	10	7
**aDI+ only**	0	0	7	7	13	10

The results for each different isotype in this table should be considered separately. Thus, a patient in the cell marked aCL+ only in the IgA aPL column does not have IgA aβ_2_GPI or IgA aDI but may have IgG or IgM antibodies to those antigens. Overall, every patient in this Table tests positive for at least one of IgG aCL, IgG aβ2GPI, IgM aCL, IgM aβ2GPI or LA. Ten LA-positive patients tested negative in all nine aPL assays, and are not included in this table.

* Inclusive of all APS patients, with or without LA positivity.

^†^ LA-positive APS patients only.

**Table 6:** Comparison of hazard ratios for APS, thrombosis and pregnancy morbidity in aCL and/or aβ_2_GPI positive subjects in the absence or presence of aDI.

	aCL/aβ_2_GPI+(aDI-)*	aCL/aβ_2_GPI+(aDI+)^†^
***IgG aPL*:**		
**No. of subjects (no. of APS)**	84 (43)	52 (45)
**Association with:**		
**APS**^‡^	**11.5 (6.3–21.0)**	**36.9 (17.7–76.9)**
**Thrombosis**^§^	**3.2 (1.1–9.1)**	**4.0 (1.4–11.2)**
**Pregnancy morbidity∞**	0.3 (0.1–1.0)	0.2 (0.1–0.9)
***IgM aPL*:**		
**No. of subjects (no. of APS)**	19 (12)	33 (29)
**Association with:**		
**APS**^‡^	**7.3 (3.0–17.5)**	**21.3 (9.1–50.4)**
**Thrombosis**^§^	1.4 (0.4–4.5)	2.3 (0.9–5.8)
**Pregnancy morbidity∞**	0.8 (0.2–3.2)	0.7 (0.3–2.0)
***IgA aPL*:**		
**No. of subjects (no. of APS)**	62 (15)	38 (32)
**Association with:**		
**APS**^‡^	**5.0 (2.7–9.2)**	**24.8 (12.3–49.9)**
**Thrombosis**^§^	1.1 (0.4–2.9)	**3.6 (1.4–9.1)**
**Pregnancy morbidity∞**	0.4 (0.1–1.4)	0.2 (0.1–0.7)

Hazard ratios (95% CI) shown for APS, thrombosis and pregnancy morbidity. Statistically significant associations are highlighted in bold.

*aCL/aβ_2_GPI+(aDI-) group includes double aCL/aβ_2_GPI and single aCL or aβ_2_GPI positives.

^†^ aCL/aβ_2_GPI+(aDI+) group includes triple positives and double aCL/aDI or aβ_2_GPI/aDI positives.

^‡^ Analysis inclusive of all subjects (n = 430). For all significant (bold) associations, p≤0.001.

^§^Analysis inclusive of APS patients only (n = 111). Significant (bold) associations, IgG aCL/aβ_2_GPI+(aDI-) p = 0.027; IgG aCL/aβ_2_GPI+(aDI+) p = 0.008; IgA aCL/aβ_2_GPI+(aDI+) p = 0.007.

^∞^ Analysis inclusive of female APS patients only (n = 93).

Of 430 subjects, 136 were positive for IgG aCL/aβ_2_GPI, 52 of which were also IgG aDI-positive; 52 subjects had IgM aCL/aβ_2_GPI of which 33 were IgM aDI-positive; and 100 subjects had IgA aCL/aβ_2_GPI of which 38 were IgA aDI-positive. For all isotypes, the presence of aDI increased the HR for APS by approximately 3-fold for IgG and IgM, and 5-fold for IgA (Table 6).

The same approach was applied to the group of 111 patients with APS in order to establish the HR for thrombosis or pregnancy morbidity associated with the aCL/aβ_2_GPI+(aDI-) and aCL/aβ_2_GPI+(aDI+) serological profiles (Table 6). Both IgG aCL/aβ_2_GPI+(aDI-) (HR for thrombosis 3.2, 95%CI 1.1–9.1) and IgG aCL/aβ_2_GPI+(aDI+) (HR for thrombosis 4.0, 95%CI 1.4–11.2) were associated with VT. No significant associations were seen for IgM or IgA serological profiles, except that the addition of IgA aDI positivity tripled the HR for thrombosis and converted it from non-significant (HR for thrombosis in aCL/aβ_2_GPI+(aDI-) subjects 1.1, 95%CI 0.4–2.9) to significant (HR for thrombosis in aCL/aβ_2_GPI+(aDI+) subjects 3.6, 95%CI 1.4–9.1). PM was not associated with either the aCL/aβ_2_GPI+(aDI-) or aCL/aβ_2_GPI+(aDI+) profile (Table 6). These findings are in agreement with results from individual regression analyses, where we determined the association of each of the nine assays with VT or PM and identified moderate positive associations between IgG aCL, aβ_2_GPI, aDI, and IgA aDI with VT but not PM (data not shown). Of interest, LA positivity alone could not discriminate between thrombotic or obstetric complications in our APS cohort (for VT, HR 1.9, 95% CI 0.7–5.1; for PM, HR 0.8, 95% CI 0.3–2.1, p>0.05).

## Discussion

In this study, we performed nine different assays using sera from 430 subjects. This large dataset allows the first rigorous comparison of IgG, IgM and IgA aCL, aβ_2_GPI and aDI in patients with APS, SLE and healthy controls. We confirm the importance of IgG aCL and IgG aβ_2_GPI tests, which had the highest sensitivity for APS and were strongly associated with LA positivity. We show that IgA aβ_2_GPI are strongly associated with APS and are more common in our cohort than IgM aβ_2_GPI, and demonstrate that aDI of all three isotypes are associated with APS with high specificity. Importantly, in subjects known to be positive for any isotype of aCL and/or aβ_2_GPI, the additional finding of aDI positivity increases the likelihood of APS by between three and five times. Finally, we report that positivity for IgG or IgA aDI increases the strength of association between aCL/aβ_2_GPI and thrombotic manifestations in APS.

While the pathogenicity of IgG aβ_2_GPI is well characterised both *in vivo* and *in vitro* [6–9], IgA aβ_2_GPI have been far less studied in comparison [4, 5]. In a recent comprehensive review, an international group of experts analysed published and unpublished data on IgA aCL and aβ_2_GPI, highlighting the low quality of the data, variability of results and that many studies had been restricted to patients with SLE [41]. Due to lack of a substantial body of evidence, IgA aPL are not included in current APS diagnostic criteria [1], yet some published guidelines suggest testing for IgA (particularly aβ_2_GPI) in patients who are IgG/IgM- and LA-negative but in whom APS is suspected [42]. Our data suggest that measurement of IgA aβ_2_GPI and aDI would be more valuable than measuring IgA aCL in patients with suspected APS, and we also note that IgA aβ_2_GPI are more commonly positive in our APS cohort compared to IgM aβ_2_GPI (Table 2). To confirm the validity of our IgA aβ_2_GPI results, we compared our IgA aβ_2_GPI test with the equivalent commercial test from Inova Diagnostics (QUANTA-Lite β_2_GPI IgA assay). We tested a total of 32 serum samples– 13 APS; 8 SLE/no APS; and 11 healthy controls—in both our in-house and the Inova assays, and found that the two assays showed very good quantitative (Spearman’s r = 0.9, p<0.0001) and qualitative agreements (93.75% of the observations agreed in terms of being positive or negative, *kappa* = 0.875).

The ability of human plasma purified β_2_GPI to recognize aPL when immobilised on a plastic surface relies on its conformation and is a significant obstacle towards standardising aβ_2_GPI tests across laboratories. In contrast, we and others have successfully used recombinant human DI expressed in bacteria [38], baculovirus [16], and synthetically [43] to measure IgG aDI antibodies [15–22, 43–45], and thus DI could potentially be a more reliable source of antigen compared to whole β_2_GPI. A number of different assays have been reported for measuring aDI (reviewed in [41]). Thus far, the most convincing published evidence arises from use of an assay dependent on comparing binding to DI on hydrophobic and hydrophilic plates [19, 21], while we have employed our in-house single-plate solid-phase assay. Detecting aPL against a small part of a protein such as DI (~7kDa) is challenging however, and thus innovative approaches should be implemented to improve both the simplicity and sensitivity of any test aimed at measuring aDI in the clinical setting. New detection methods could help achieve this goal. Inova Diagnostics developed a chemiluminescent immunoassay (CIA) for IgG aDI that recently received clearance by the U.S. Food and Drug Administration for use in autoimmune disease testing, and report a sensitivity of 85% in a cohort of 144 patients with APS, compared to 0.5% and 14% for 200 healthy and 72 infectious disease controls respectively [14]. In a small study of 39 patients with APS and 77 disease and healthy controls, the IgG aDI CIA had a sensitivity of 36%, while an IgG aβ_2_GPI CIA had a sensitivity of 46% [44]. Likewise, our IgG aβ_2_GPI assay proved to have higher sensitivity for APS compared to aDI (Table 3). Moreover, positivity for IgG, IgM and IgA aDI was strongly associated with APS to a similar or better level than the corresponding aCL assays, though not as strongly as with aβ_2_GPI (Table 4). The apparent superiority of aβ_2_GPI assays is likely due to the presence of antibodies that target DII-V. Indeed, we recently utilised the Inova IgG aDI CIA and a further prototype CIA to measure IgG aβ_2_GPI against domains IV-V (aDIV-V), demonstrating that both aDI and aDIV-V can be detected in the blood of aβ_2_GPI-positive subjects. Importantly however, aDI were more frequently found in patients with systemic autoimmune disease compared to asymptomatic aPL carriers [15]. Of note, we reported good qualitative and quantitative agreement between our IgG aDI ELISA and the Inova CIA, as well as similar discrimination for APS compared to controls [41, 46].

In line with recently published studies [15, 45], we also found that aDI were more common in APS patients who were aCL, aβ_2_GPI and LA-positive (Table 5). Interestingly, we identified two LA-positive APS patients with low IgG aCL and aβ_2_GPI (patient 1, 20GPLU and 8GBU; patient 2, 27GPLU and 12GBU) but high IgG aDI (>50GDIU). One of these patients also had high IgM aDI (>100MDIU) despite low IgM aCL (20MPLU) and IgM aβ_2_GPI (21MBU). Therefore, although rare, there are patients with APS with high aDI activity but low or negative classical IgG/IgM aPL activity, and in these cases aDI tests could certainly provide additional information to current tests available. Of relevance, we recently reported the presence of IgG aDI in 3 out of 40 ‘seronegative’ APS patients, who fulfilled clinical but not serological APS criteria [47], further suggesting that detecting aDI could complement current criteria tests.

Our results concur with the largest published study for IgG aDI in 442 aβ_2_GPI-positive subjects (of which 82% had APS), underlining the added value of measuring IgG aDI as well as aCL/aβ_2_GPI in relation to increased risk of VT [21]. We additionally report that IgA aDI are of similar value for determining thrombotic risk (Table 6). Unlike the study of de Laat *et al* [21] however, we did not find any benefit of adding IgG aDI in terms of an association with PM. This dissimilarity may have arisen because the majority of our female APS patients (67%) who suffered pregnancy complications did not have a thrombotic history, compared to 42% in the original study. A very recent study also concluded that IgG aDI are associated with thrombosis but not pregnancy loss in a cohort of 65 IgG aβ_2_GPI-positive subjects [45].

## Conclusions

Based on our current findings and other groups’ published reports, we consider aDI tests as a useful additional test rather than a replacement for tests using whole β_2_GPI, since the latter would also pick up aβ_2_GPI directed against other domains. Considering their pathogenic role, detecting IgG aDI may allow for risk stratification in established APS and help in the diagnosis of suspected APS, while the prevalence and clinical association of IgA aDI in APS requires further clarification. Moreover, prospective studies are imperative to determine if IgA aβ_2_GPI and IgG/IgA aDI have prognostic power. We are currently completing such a study in early samples from >500 patients with SLE, a proportion of whom later developed thrombosis or obstetric complications, and are performing longitudinal tests in order to establish if aPL levels remain constant or change before, near to and after an APS-related clinical event.
